# Data on hydrogen isotopes yield from Pd under thermal, electric current, radiation and UV stimulations

**DOI:** 10.1016/j.dib.2019.104850

**Published:** 2019-11-21

**Authors:** Yu.I. Tyurin, V.S. Sypchenko, N.N. Nikitenkov, Hongru Zhang, I.P. Chernov

**Affiliations:** Tomsk Polytechnic University, 30 Lenin Avenue, Tomsk 634004, Russia

**Keywords:** Hydrogen isotopes, Surface, Non-equilibrium processes, Atomic migration, Palladium, Radiation-and thermo-stimulated hydrogen release

## Abstract

Data on the hydrogen isotopes (H, D) yield of Pd with linear heating: a) by the accelerated electrons beam with energy up to 35 KeV, b) by joule heat of AC (50 Hz) through samples, c) by external coaxial metal furnace (stainless steel), d) in quartz vacuum cell are presented and e) UV stimulation during thermal heating (the research article [2]).

The highest temperature position of the maximum hydrogen isotopes intensity release corresponds to the samples heating in a metal vacuum cell by external coaxial furnace. The lowest temperature position of the maximum intensity hydrogen isotopes release corresponds to the heating by accelerated electrons beam. The difference in these positions of the maximum is ΔТ ≈ 300°С. Shift of maxima position in the hydrogen and deuterium release into the low-temperature region is significant (ΔТ ≈ 50–100°С) for the Pd sample when metal are heated by electric current or in a quartz vacuum cell compared to their heating in a metal vacuum cell and under UV stimulation during thermal heating.

**Table:** Specifications Table

Subject area	Physics
More specific subject area	Hydrogen–solid state matter systems under external influence
Type of data	text file, graph
How data was acquired	A plant for studying radiation and thermal desorption of gases from inorganic materials. Instruments Exp Tech 2009; 52: 865–70. doi:10.1134/S0020441209060207 [3]. Additionally: a DTH-125-1 mercury lamp; Automated system “Project” for recording temperature spectra in the “TXT” format with data transmission to the table of the Origin Pro package (produced at Tomsk Polytechnic University); mass spectrometry Stablil-Ion Granwill-Philips (835 VQM), RHEN 602 hydrogen analyzer.
Data format	Raw and analyzed data
Experimental factors	Saturate sample of palladium Pd (0.9999) with hydrogen and deuterium used. Saturation to high H and D concentrations by the Siverts's method and cathodic saturation by H and D in a 0.1–1 M solution of H_2_SO_4_ + H_2_O or D_2_SO_4_ + D_2_O are used. Installation for thermo-stimulated gas release and radiation-stimulated heating of the samples by accelerated electrons beam with energy 10–35 KeV, current density 1–100 μA cm^−2^. Device OUFK-01 for UV stimulation.
Experimental features	The data performed in a high-vacuum installation [3] (residual pressure P_res_<10^−6^ Torr). Heating of samples from 20 to 1000°C with a linear rate from 0.1 to 5°C· s^−1^. For photo activation of samples was used ultraviolet light (UV) of a DTH-125-1 mercury lamp, 100 <λ < 400 nm, W ≈ 1.5W/m^2^, 1600 lm. The date of thermally stimulated gas release (TSGR) carried out using external linear heating of samples by coaxial furnace in evacuated quartz or metal cells.
Data source location	Tomsk, Russia
Data accessibility	Data are included in this article
Related research article	Yu.I. Tyurin, V.S. Sypchenko, N.N. Nikitenkov*, Hongru Zhang, I.P. Chernov. Comparative study of the hydrogen isotopes yield from Ti, Zr, Ni, Pd, Pt at thermal, electric current and radiation heating of these metals, Int J Hydrogen Energy, DOI: 10.1016/j.ijhydene.2019.05.185.

**Table:** 

**Value of the Data** • The data on the features of the hydrogen release from metals by the heating method give a notion about the mechanisms of stimulating the release of hydrogen by electromagnetic fields and ionizing radiation in the subthreshold region. • Data on the features of hydrogen release during thermal and radiation heating for metals, which are forms solid solutions with hydrogen isotopes are relevant to prevent hydrogen embrittlement of construction materials of atomic, thermonuclear and hydrogen energy. • Data on the features of the stimulating processes the hydrogen release from metals at heated by joule heat can be useful to researchers to improve the stability of the products of microelectronics. • Data on the features of the hydrogen isotopes release during thermal heating and UV stimulation are relevant in the photoactivation of the metal hydrides decomposition. UV exposure accelerates the desorption processes of hydrogen molecular formed by H and D atoms diffusing from volume on the metal surface. It is important to create hydrogen storage devices and develop methods for accelerating physical processes at interphase boundaries in metal – hydrogen systems [1].

## 1 Data

This brief article describes the data upon the stimulation hydrogen isotopes (H,D) yield from Pd at equilibrium and non-equilibrium heating of the samples with help: a) external coaxial furnace, b) the Joule's heat of electric current, c) accelerated electrons beam, d) weak electromagnetic fields and e) UV stimulation during thermal heating.

**Fig. 1:**
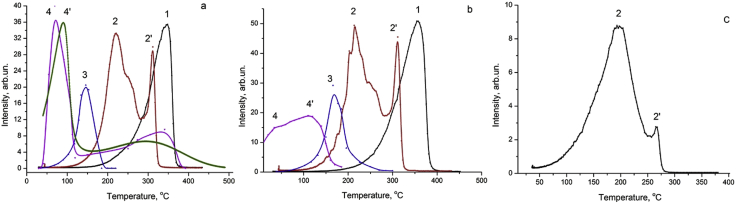
The hydrogen isotopes release ((а) – H_2_, (b) – (D_2_)) from Pd at the linear heating: in stainless steel vacuum cell T_max_(H_2_) = 355 °C, T_max_(D_2_) = 360 °C (curves 1 а, б); in quartz vacuum cell T_max_(H_2_) = 220 °C (peak 2а), T_max_(H_2_) = 312 °C (peak 2′а), T_max_(D_2_) = 217 °C (peak 2б), T_max_(D_2_) = 311°C (peak 2′б); T_max_(D_2_) = 207 °C (peak 2с), T_max2_(D_2_) = 270°C (peak 2′с) (curves 2 а,b,с); heating by AC: T_max_(H_2_) = 150 °C, T_max_(D_2_) = 165 °C (curves 3 а,б); radiation-stimulated hydrogen release (curves 4 a,b); analytical approximation of the experimental curve 4a (криϑая 4а′).

Fig. 1a and b (curves 1–3) shows the dependences of the hydrogen and deuterium yield from palladium in the linear heating modes of sample, during the heating in stainless steel and quartz vacuum cells. Fig. 1a and b (curve 4) shows the dependences of the intensity hydrogen release from palladium in the linear heating mode of the sample and in the sample radiative heating with an accelerated electron beam. Fig. 1c shows the dependences of the hydrogen and deuterium intensity yield from palladium strongly saturated by deuterium in the linear heating modes of sample, during heating in quartz vacuum cell.

**Fig. 2:**
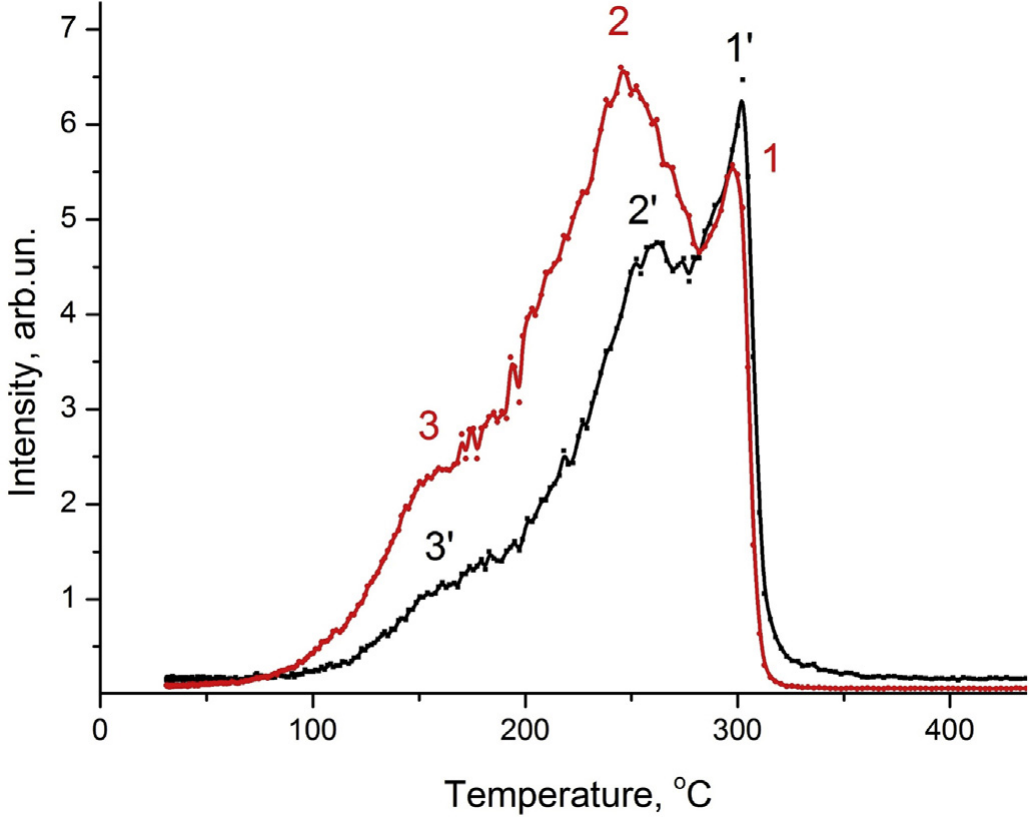
Hydrogen isotopes release: H (1′-3′) and D (1–3) from the closed loop of Pd at the linear heating mode (heating rate = 1°C·s^−1^) by a quartz coaxial furnace (j = (30–70) А сm ^– 2^) in a quartz vacuum cell: 1, 1′ peaks are the thermal stimulation contribution: T_max1_ (D_2_), T_max1_ ‘(H_2_) = 300 °C; 2, 2′ peaks are the electromagnetic field contribution: T_max2_ (D_2_) = 246 °C, T_max2_’ (H_2_) = 262 °C; 3, 3′ peaks are the induced electric current contribution: T_max3_ (D_2_), T_max3’_ (H_2_) = 16 °C. Electrolytic saturation of Pd: D_2_SO_4_ (0.5 M), t = 90min, I = 100mA, 02.15.2018.

Three peaks are observed when a closed coil of Pd-D,H is heated in a coaxial quartz furnace in a quartz cell, Fig. 2. The additional maximum at (155 ± 50C) (Fig. 2) correlates with the position of the AC-maximum (curve 3, Fig. 1a and b).

**Fig. 3:**
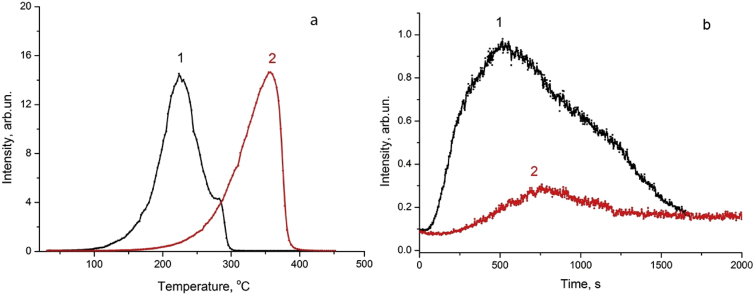
Deuterium release from palladium at linear heating: (a) – heating rate is 1 °C s^−1^ with simultaneous irradiation of the palladium surface with UV (curve 1) and without UV irradiation (curve 2); (b) – heating rate is 0.1 °C/s, sample temperature from 25 to 65 °C (0˂t˂650 s) with simultaneous irradiation of the palladium surface with UV (1b) and without UV (2b); (1b) – t_max_ UV = 560 s, (2b) t_max_ T = 770 sec; sample size: 0.05 × 3 × 25 mm. Electrolytic saturation of Pd in D_2_SO_4_ (1,0 M) + in D_2_O, t = 2h, I = 70mA cm^−2^.

Fig. 3 shows the temperature dependence of the intensites of the hydrogen and deuterium yields from Pd at linear heating of the sample and UV stimulation.

Analytical approximations indicate possible mechanisms for stimulating radiation, UV and dynamic electromagnetic processes (curve 4′, Fig. 1a) [2].

## 2 Experimental design, materials and methods

### 2.1 Experimental installation

The data were received in a high-vacuum installation with oil-free pumping (P_res_˂10^−6^ Torr), the gases emitted from the materials were recorded by a mass spectrometer [3]. The data of thermally stimulated gas release (TSGR) carried out using external linear heating of samples by coaxial quartz furnace. Samples placed in evacuated quartz or metal cells. To heat the filament of furnace wound on a quartz tubes as solenoid 50 Hz (AC, Joule's heat), current density 120–420 A cm^−2^, the solenoid turns is 10 cm^−1^, the length of the furnace are 10 cm. The sample temperature controlled by chromel-alumel thermocouple. The error in determining the temperature positions of the peaks (T_max_) in the H and D isotopes thermal spectra was ±5 °C. The heating regimes is set by the “Project” computer program and allows heating of samples from 20 to 1000°C with a linear velocity from 0.1 to 5°C s^−1^. The system is able to track the intensities of up to six lines of mass spectra of gases released from the sample during heating and (or) irradiation.

For radiation-stimulated heating of the samples by an electron beam, an electron gun with a hot cathode and a system of magnetic focusing of the accelerated beam was used. The energy of 10–30 KeV, the beam varies from 1 to 100 μA cm^−2^ The electron beam current was determined directly from the sample. The heating element allows to maintain a constant temperature on the sample or to perform additional linear heating of the sample during irradiation.

For the photoactivation of the samples, the UVT-125-1 mercury lamp was used, 100 <λ < 400 nm, W ≈ 1.5 W/m^2^, 1600 lm.

In addition, heating was performed by passing an electric current (AC 50 Hz) directly through the sample – Joule's heat – also carried out by a programmable heating device. The hydrogen concentration in the samples measured with the RHEN 602 hydrogen analyzer from LECO Corporation.

### 2.2 Selection of sample

The Pd has not chemical bonds “metal–hydrogen” formation, but forming solid solutions with hydrogen isotopes. Gas emission from pre-saturated with hydrogen and deuterium palladium (0.9999) investigated in the linear heating mode. The geometry of the Pd samples was chosen in the form of plane-parallel plates of 0.05 × 3 × 25 (thermal, thermal + UV and Joule's heating), 0.2 × 10 × 10 mm (radiation heating) sizes. The thermal and Joule's heating rate was 1°C s^−1^, the radiation heating rate reached 10 ÷ 15°C·s^−1^.

### 2.3 Saturation of samples with hydrogen and deuterium

For saturating samples of Pd with hydrogen and deuterium were used: a) the Siverts's method in the PCI installation of the Gas Reaction Controller; b) the electrolytic (cathodic) saturation in a 0.1–1 M solution of H_2_SO_4_ + H_2_O or D_2_SO_4_ + D_2_O from 2 to 72 hours at current density of 2–200 mA cm^−2^ under normal conditions.

This also ensures a relatively uniform distribution of hydrogen in the samples volume at low electrolysis current densities (2 mA cm^−2^) and long time (≈10^5^ s) saturation.

### 2.4 Data study

We found that the positions of the peaks of thermal and gas release of hydrogen and deuterium from palladium during heating of samples in a metal cell, in a quartz cell, electric current (Fig. 1, Fig. 2),UV, UV + heating in a quartz cell (Fig. 3) and a beam of accelerated electrons (Fig. 1) differ significantly.

We found that the positions of the peaks of thermal and gas release of hydrogen and deuterium from palladium during heating of samples in a metal cell, in a quartz cell, electric current (Fig. 1, Fig. 2), heating with a stimulating effect of ultraviolet mercury lamp (Fig. 3) and a beam of accelerated electrons (Fig. 1a and b, curves 4,4′) differ significantly.

The conditions under which the data presented in Fig. 1 were follows. The area of AC density change during heating in the quartz furnace spiral was (120 ÷ 420) A cm ^– 2^ and AC through a sample Pd-D,H was (40–120) A ∙ cm^– 2^. AC voltage used to heat the sample of 3a, b, c was (1–15) V. Conditions of cathode saturation for curves 1–3, Fig. 1a and b was 0.5 M, t = 0.5 h, j = 100 mA cm^−2^, for curve 4, Fig. 1b it was 0.1 M, t = 20 h, j = 9 mA ∙ cm^−2^ and for Fig. 1c it was 1 M, t = 2h, j = 70 mA cm^−2^. Sizes of samples were 0.05 × 3 × 25 mm (Fig. 1a–c, curves 1–3) and 0.2 × 10 × 10 mm (Fig. 1a and b, curve 4). Electron beam energy was E = 35 keV (Fig. 1a, curve 4), 20 keV (Fig. 1b, curve 4). Electron beam current density was j = 75 μA cm^−2^ (Fig. 1a, curve 4) and j = 20 μA (Fig. 1b, curve 4). Heating rate were 1 °C ∙ s^−1^ (Fig. 1a–c, curves 1–3), 15 °C s^−1^ (Fig. 1a, curve 4), 0.4 °C s^−1^ (Fig. 1b, curve 4).

The shift of the maxima position temperature of the releasing hydrogen isotopes flux density during thermal heating in metallic stainless steel and heating with alternating electric current (AC, 50 Hz) for palladium is ΔТ = (198 ± 5) °C. The shift in the maxima position when heated by electric current and heated in a quartz cell by an external coaxial furnace is for the first maximum ΔТmax1 = (63 ± 5) °C. The position of the low-temperature maximum of hydrogen release and deuterium when heated palladium in quartz cell at (217 ± 5) °C is correlates with the position of the peak (157 ± 7) °C when palladium heated by Joule heat. Nevertheless, the high-temperature peak (311 ± 5) °C is correlates with the peak (355 ± 5) °C when the palladium is heated in the metal cell (see Fig. 1a and b). This correlation is associated with the effect of electromagnetic fields on diffusion processes in metals, where hydrogen is not fixed by a strong chemical bond, but is located in the internodes in the H^+^, D^+^ states close to ionic. When palladium is heated by a beam of accelerated electrons, the shift in position with respect the maximum of the equilibrium yield of hydrogen isotopes (cf. curves 1 and 4, Fig. 1a and b) reaches 300^°^C.

An increase of the hydrogen isotopes concentration in samples increases the contribution in the low-temperature (210 °C) non-equilibrium component of the hydrogen isotopes release (Fig. 1c). Really, the intensity ratio of the lines is K1 = J (210 °C)**/**J (275 °C) ≈3.6 (Fig. 1c), K2 = J (217 °C)**/**J (311 °C) ≈0.9, (Fig. 1b), so K1**/**K2 = 4 vol ratio of introduced hydrogen is H (Fg.1c)**/**H (Fig. 1b) = 5.6.

Three peaks are observed when a closed coil of Pd-D,H is heated in a coaxial quartz furnace in quartz cell (Fig. 2). The additional maximum at (155 ± 50С) (Fig. 2) correlates with the position of the AC-maximum (curv.3, Fig. 1a and b).

Heating samples of Pd-D, H in the form of a closed turn in a vacuum quartz cell combines the effects of thermal heating in a quartz cell and heating with electric current (Fig. 2). This allows you to implement a fairly simple method of detecting the stimulating effect of electric current on the hydrogen release from metals.

The acceleration of the hydrogen and deuterium release from saturated palladium is also observed with linear heating to 65 °C (at a rate of 0.1 °C**/**s^−1^) with simultaneous irradiation of the surface with UV-light of a mercury lamp (Fig. 3a and b).

The temperature position in the maximum hydrogen and deuterium release rates at comparable heating rates for Ni similar to Pd samples and decrease in the following order: heating by external electric furnace in metal cell → in quartz cell → by Joule's heat (displacement reached of ΔT = 100–200 °C). However, for heating by the accelerated electrons beam one has ΔТ≈350 ^°^C [2].

Titanium, like zirconium, belongs to the IVB group, V period of the periodic table. For zirconium, as for titanium, there is no significant difference in the position of the temperature maxima of the intensity of the hydrogen isotopes yield with 3 methods of heating the samples by joule heat of AC (50 Hz) through samples, external coaxial furnace samples in metal (stainless steel) and quartz vacuum cells [2].

For linear heating of titanium and zirconium with simultaneous irradiation with an electron beam, there is a noticeable shift (ΔT = (365 ± 5) °C) in the position of maximum yield during the electron beam stimulation in the low-temperature region, compared with the maximum in the equilibrium thermal heating in the metal vacuum cell [2].

Was determined that the isotope effect (differences in the H and D releases at different ways to stimulate the release of hydrogen isotopes) is relatively weak, but there is a tendency to an increase in temperature displacements for H-isotope when there is a non-equilibrium component in the input energy (material depending) [[2], [3], [4], [5]].
